# Children with ADHD Symptoms Have a Higher Risk for Reading, Spelling and Math Difficulties in the GINIplus and LISAplus Cohort Studies

**DOI:** 10.1371/journal.pone.0063859

**Published:** 2013-05-27

**Authors:** Darina Czamara, Carla M. T. Tiesler, Gabriele Kohlböck, Dietrich Berdel, Barbara Hoffmann, Carl-Peter Bauer, Sibylle Koletzko, Beate Schaaf, Irina Lehmann, Olf Herbarth, Andrea von Berg, Bertram Müller-Myhsok, Gerd Schulte-Körne, Joachim Heinrich

**Affiliations:** 1 Max Planck Institute of Psychiatry, Munich, Germany; 2 Munich Cluster for Systems Neurology (SyNergy), Munich, Germany; 3 Helmholtz Zentrum München, German Research Center for Environmental Health, Institute of Epidemiology I, Neuherberg, Germany; 4 Ludwig-Maximilians-University of Munich, Dr. von Hauner Children’s Hospital, Division of Metabolic Diseases and Nutritional Medicine, Munich, Germany; 5 Marien-Hospital Wesel, Department of Pediatrics, Wesel, Germany; 6 IUF Leibniz Research Institute for Environmental Medicine and Medical Faculty, Heinrich Heine University of Düsseldorf, Düsseldorf, Germany; 7 Technical University of Munich, Department of Pediatrics, Munich, Germany; 8 Ludwig-Maximilians-University of Munich, Dr. von Hauner Children’s Hospital, Division of Paediatric Gastroenterology and Hepatology, Munich, Germany; 9 Medical Practice for Pediatrics, Bad Honnef, Germany; 10 Helmholtz Centre for Environmental Research – UFZ, Department for Environmental Immunology, Leipzig, Germany; 11 University of Leipzig, Faculty of Medicine, Environmental Medicine and Hygiene, Leipzig, Germany; 12 Department of Child and Adolescent Psychiatry, Psychosomatics and Psychotherapy, Ludwig-Maximilians-University of Munich, Munich, Germany; Western University of Health Sciences, United States of America

## Abstract

Attention-deficit/hyperactivity disorder (ADHD) and dyslexia belong to the most common neuro-behavioral childhood disorders with prevalences of around 5% in school-aged children. It is estimated that 20–60% of individuals affected with ADHD also present with learning disorders. We investigated the comorbidity between ADHD symptoms and reading/spelling and math difficulties in two on-going population-based birth cohort studies. Children with ADHD symptoms were at significantly higher risk of also showing reading/spelling difficulties or disorder (Odds Ratio (OR) = 2.80, p = 6.59×10^−13^) as compared to children without ADHD symptoms. For math difficulties the association was similar (OR = 2.55, p = 3.63×10^−04^). Our results strengthen the hypothesis that ADHD and learning disorders are comorbid and share, at least partially, the same underlying process. Up to date, it is not clear, on which exact functional processes this comorbidity is based.

## Introduction

Attention-deficit/hyperactivity disorder (ADHD) belongs to the most common neuro-behavioural childhood disorders [1] with a world-wide prevalence of 5.3% [2]. According to DSM-IV (Diagnostic and Statistical Manual of Mental Disorders) [3] it is characterized by two core symptoms which co-occur: inattention and overactivity-impulsiveness at home as well as in school environment. 75% of affected children are male [4].

Several environmental factors were suggested to increase the risk for ADHD. Among them, time spent in front of the TV [5], single parent headed households [6], parental education [7], maternal age at birth [7], and maternal smoking were reported [8]–[10]. Up to now, genome-wide association studies suggested markers which exhibit only low effect sizes on ADHD susceptibility [1].

Interestingly, ADHD is associated with other neurological and psychiatric disorders. More than 60% of individuals suffering from ADHD present with one ore more comorbid disorders [11] like oppositional defiant disorder, conduct disorder, depression, anxiety and tic disorders. 20–60% of children exhibiting ADHD are also affected by learning disorders [12], [13].

Two of the most common learning disorders are dyslexia and dyscalculia with prevalence rates of about 5% in school-aged children [14], [15]. Whereas dyslexia is more frequent in boys than in girls [16], no sex differences in prevalence rates have been reported for dyscalculia [17]. Dyslexic children suffer from difficulties in learning to read and spell which are not caused by neurological deficits, impairments in general intelligence or inadequate education. Dyscalculia is characterized by deficits in the acquisition of numerical abilities and calculation skills which are not due to low intelligence, inadequate schooling or sensory impairments [15].

To date, only few studies have examined the comorbidity between ADHD symptoms and reading/spelling and math difficulties. Especially the relationship between ADHD and dyscalculia has not yet been studied in detail yet.

We therefore investigated whether children with ADHD symptoms have a higher risk for also being affected with reading/spelling and math difficulties in a large population-based sample of over 4,000 children aged 10 years, in which also possible confounding factors like maternal smoking had been collected.

## Methods

### Ethics Statement

The LISAplus (The Influence of Life-style factors on the development of the Immune System and Allergies in East and West Germany plus the influence of environment and genetics) and the GINIplus (German Infant Study on the influence of Nutrition Intervention plus environmental and genetic influences on allergy development) studies were approved by the local ethics committees (Ethikkommission der Bayrischen Landeärztekammer, Ethikkommission an der Medizinischen Fakultät der Universität Leipzig, Ärztekammer Nordrhein) and written consent was obtained from the parents of all study participants.

### Study Design

LISAplus and GINIplus are ongoing population-based birth cohort studies.

For the LISAplus study, the parents of neonates admitted to maternity hospitals in four German cities (Munich, Leipzig, Wesel and Bad Honnef) were contacted and 3,097 healthy full-term neonates were recruited in the study between December 1997 and January 1999. Screening, recruitment and exclusion criteria were described elsewhere [18], [19]. Follow-up time points were at the ages of 0.5, 1, 1.5, 2, 4, 6 and 10 years.

For the GINIplus study, a total of 5,991 healthy full-term infants born between September 1995 and June 1998 in two regions of Germany (urban Munich and rural Wesel) were recruited. The study population is subdivided into an interventional (n = 2,252) and an observational group (n = 3,739). Children with family history of allergy were assigned to the intervention group and the observational subgroup comprises children with no family history of allergic disease and children whose parents refused to participate in the intervention trial. A description of the study design has been published previously [20]. Follow-up questionnaires on child’s health were administered to the parents at 1, 2, 4, 6 and 10 years after birth.

In both studies, reading/spelling disorders or problems were assessed by questionnaire at the 10-year follow-up. ADHD symptoms at the age of 10 years were measured by the German parent-reported version of the Strengths and Difficulties Questionnaire (SDQ) [21]–[24].

### Questionnaire Data

In GINIplus, the presence of a reading/spelling disorder or reading/spelling problems was assessed by one question each. Children, whose parents answered that their child presented with reading/spelling problems as well as with reading/spelling disorder were grouped into the more severe category of reading/spelling disorder, i.e. both categories are disjunct. In LISAplus, parents were asked a single question, i.e. whether their child suffered from reading/spelling disorder or reading/spelling problems.

In GINIplus and in LISAplus, parents were also asked via a single question, whether their child suffered from dyscalculia or had problems in mathematics.

In order to adjust the multivariable regression models in the analysis for covariates that might lead to confounding of the results, we included gender, age, study (GINIplus or LISAplus) and study center (Munich, Leipzig, Bad Honnef or Wesel) as basic characteristics of the child. Parental education, single-headed households, maternal age at birth, time which the child spent in front of a screen and pre- and/or postnatal environmental tobacco smoke (ETS) exposure are effect modifiers which have already been reported [5]–[10] and which were also available in LISAplus and GINIplus. Parental educational level was measured on a scale of ‘low’ (both parents with less than 10 years of schooling) over ‘medium’ (at least one parent with 10 years of schooling) to ‘high’ (at least one parent with more than 10 years of schooling). We further included maternal age at birth (more or equal to 30 years or younger) and single parent status at the child’s age of 10 years. The time which the child usually spent in front of a screen (e.g. television, computer, …) during the tenth year of life was categorized in ‘low’ defined as less than one hour per day in summer and less than two hours per day in winter compared to ‘high’. Pre- and/or postnatal ETS exposure of the child at home was assessed by a four-category variable (‘never’ ‘only postnatal’, ‘only prenatal’ and ‘pre- and postnatal’). A child was defined to be prenatally exposed if the mother reported active smoking in at least one trimester during pregnancy. Children who were reported to be exposed to ETS at home up to 10 years were classified as postnatally exposed.

Current methylphenidate treatment of ADHD symptoms was assessed by questionnaire at the 10-year follow-up.

### Outcome Measure

ADHD symptoms were assessed by the hyperactivity/inattention subscale of the SDQ [21]–[24], an internationally disseminated and validated screening questionnaire to assess mental and behavioral strengths and difficulties in 3- to 16-year-olds. The five items for the hyperactivity/inattention subscale are “restless, overactive, cannot stay still for long”, “constantly fidgeting or squirming”, “easily distracted, concentration wanders”, “thinks things out before acting”, “sees tasks through to the end, good attention span”. Every item is rated on a three-point scale: ‘not true’ (0), ‘somewhat true’ (1) and ‘certainly true’ (2). Positively worded items are reverse-scored. The score for the ‘hyperactivity/inattention’ subscale is calculated as the sum of the ratings. The children were then categorized into “normal”, “borderline” and “high” scores according to cut-off points recommended for German samples [24].

### Statistical Analysis

Individuals from both studies, GINIplus and LISAplus, were pooled into one sample. Comorbidity of either reading/spelling difficulties or math difficulties and ADHD symptoms was assessed using logistic regression. Reading/spelling and math difficulties, respectively, were used as dependent and ADHD symptoms as independent variable. Children with normal SDQ hyperactivity/inattention levels were defined as reference category, thus the logistic regression model yielded odds ratios (ORs) and significance values for the comparisons high to normal and borderline to normal SDQ hyperactivity/inattention scores.

Three different models were applied: no adjustment for covariates (dyslexia crude/dyscalculia crude), adjustment for the covariates gender, age, study and study centre (dyslexia adjusted I/dyscalculia adjusted I), additional adjustment for possible modifiers as parental education, age of mother at birth, daily TV/PC use, single parent and ETS exposure (dyslexia adjusted II/dyscalculia adjusted II). Additionally, we conducted a stratified analysis for gender and study. Statistical significance was defined by a two-sided alpha level of 5%. The results are presented as OR with corresponding 95% confidence interval (CI) and p-value. All analyses were performed using R, version 2.13.1 (http://www.r-project.org/).

## Results

Sample characteristics for the core phenotypes as well as for covariates are depicted in Table 1. 8.1% of all children presented with ADHD symptoms and 25% of those were also affected with reading/spelling difficulties. Children with ADHD symptoms were at higher risk of also being affected with reading/spelling difficulties as compared to children without ADHD symptoms (Table 2). Without adjustment for covariates, the OR for also having reading/spelling difficulties was 4.39 (p = 2.50×10^−23^) for children with borderline and 3.57 (p = 1.48×10^−22^) for children presenting with a high SDQ-score. After adjusting for gender, age, study, study center, parental education, age of mother at birth, daily TV/PC use, single parent and ETS exposure (adjusted II model), these values dropped to 3.95 (p = 7.27×10^−17^) and 2.80 (p = 6.59×10^−13^), respectively. Stratified analyses for GINIplus and LISAplus revealed the same results (data not shown).

**Table 1:** Study characteristics of children aged 10 years from GINIplus and LISAplus.

	SDQ hyperactivity/inattention category1)			
	Normal	Borderline	High	Total
Gender				
Male	2027 (81.9%)	163 (6.6%)	285 (11.5%)	2475
Female	2180 (91.4%)	95 (4.0%)	111 (4.6%)	2386
Study				
GINIplus	2782 (86.7%)	163 (5.1%)	262 (8.2%)	3207
LISAplus	1425 (86.2%)	95 (5.7%)	134 (8.1%)	1654
Study center				
Munich	2219 (87.0%)	128 (5.0%)	204 (8.0%)	2551
Leipzig	334 (84.8%)	23 (5.8%)	37 (9.4%)	394
Bad Honnef	175 (87.9%)	13 (6.5%)	11 (5.6%)	199
Wesel	1479 (86.1%)	94 (5.5%)	144 (8.4%)	1717
Parental education				
Both parents <10 yrs	256 (78.1%)	27 (8.2%)	45 (13.7%)	328
At least one parent 10 yrs	1046 (82.9%)	85 (6.7%)	132 (10.4%)	1263
At least one parent >10 yrs	2747 (88.9%)	138 (4.4%)	206 (6.7%)	3091
Age of mother				
At birth <30 yrs	1251 (83.9%)	94 (6.3%)	146 (9.8%)	1491
At birth at least 30 yrs	2924 (87.8%)	159 (4.8%)	246 (7.4%)	3329
Daily TV/PC use				
<1 h in summer & <2 h in winter	2790 (87.7%)	162 (5.1%)	230 (7.2%)	3182
> = 1 h in summer or > = 2 h in winter	1361 (84.3%)	93 (5.7%)	161 (10.0%)	1615
Single parent				
No	3714 (87.9%)	199 (4.7%)	312 (7.4%)	4225
Yes	422 (76.6%)	52 (9.4%)	77 (14.0%)	551
Exposure to tobacco smoke at home				
Never	2220 (89.4%)	108 (4.3%)	156 (6.3%)	2484
Only postnatal	1197 (84.7%)	83 (5.9%)	133 (9.4%)	1413
Only prenatal	101 (85.6%)	7 (5.9%)	10 (8.5%)	118
Pre- and postnatal	391 (77.1%)	43 (8.5%)	73 (14.4%)	507
Special training in reading/spelling				
No	3553 (89.5%)	167 (4.2%)	249 (6.3%)	3969
Yes	521 (70.8%)	81 (11.0%)	134 (18.2%)	736
Reading/spelling difficulties2)				
No	3699 (89.1%)	173 (4.2%)	280 (6.7%)	4152
Yes	365 (67.8%)	75 (14.0%)	98 (18.2%)	538
Severity of dyslexia3)				
Reading/spelling problems	191 (72.0%)	37 (14.0%)	37 (14.0%)	265
Reading/spelling disorder	79 (65.8%)	10 (8.3%)	31 (25.9%)	120
Math difficulties				
No	4000 (87.1%)	235 (5.1%)	355 (7.8%)	4590
Yes	102 (70.8%)	16 (11.1%)	26 (18.1%)	144

given numbers indicate the number of individuals with available corresponding phenotypes.

1) SDQ hyperactivity/inattention is categorized as normal (SDQ score lower than 6), borderline (SDQ score between 6 and 7) and high (SDQ score higher than 7).

2) refers to children with reading/spelling disorder as well as to children with reading/spelling problems.

3) distinction between reading/spelling problems and reading/spelling disorder was only made in GINIplus.

**Table 2:** Association results from logistic regression analysis between reading/spelling difficulties and ADHD symptoms measured by the SDQ-hyperactivity/inattention score.

SDQ hyperactivity/inattention score											
Model	Normal			Borderline				High			
	dyslexic^1^	nondyslexic^2^	OR	dyslexic^1^	nondyslexic^2^	OR[95% CI]	p-value	dyslexic^1^	nondyslexic^2^	OR[95% CI]	p-value
Dyslexiacrude	n = 365	n = 3699	1.00(reference)	n = 75	n = 173	4.39[3.28–5.88]	**2.50**×**10** ^−**23**^	n = 98	n = 280	3.57[2.75–4.57]	**1.48**×**10** ^−**22**^
Dyslexiaadjusted I^3^	n = 365	n = 3699	1.00(reference)	n = 75	n = 173	4.30[3.20–5.78]	**3.35**×**10** ^−**22**^	n = 98	n = 280	3.25[2.51–4.21]	**5.07**×**10** ^−**19**^
Dyslexiaadjusted II^4^	n = 333	n = 3298	1.00(reference)	n = 65	n = 154	3.95[2.86–5.45]	**7.27**×**10** ^−**17**^	n = 87	n = 253	2.80[2.11–3.70]	**6.59**×**10** ^−**13**^

1 refers to the number of individuals with reading/spelling difficulties for whom full phenotypic information was available and who were included in regression analysis.

2 refers to the number of individuals without reading/spelling difficulties for whom full phenotypic information was available and who were included in regression analysis.

3 adjusted for gender, age, study and study center.

4 additional adjustment for parental education, age of mother at birth, daily TV/PC use, single parent and ETS exposure.

p-values <0.05 are depicted in bold.

As gender differences in the prevalence of ADHD [4] and reading/spelling problems [16] have been reported, we conducted a sex-stratified analysis in the combined GINIplus and LISAplus sample to test if comorbidity rates differed between boys and girls. The prevalence for being affected by reading/spelling difficulties was higher in males (14.1%) than in females (8.7%). Males presenting with borderline SDQ-score had a higher risk than corresponding females (adjusted II model- males: OR = 4.11, p = 6.68×10^−12^, females: OR = 3.69, p = 3.25×10^−06^). Unexpectedly, males presenting with high SDQ-score showed a lower risk for also being affected with reading/spelling difficulties than the female group (adjusted II model- males: OR = 2.36, p = 9.15×10^−09^, females: OR = 3.34, p = 1.41×10^−05^).

**Figure 1:**
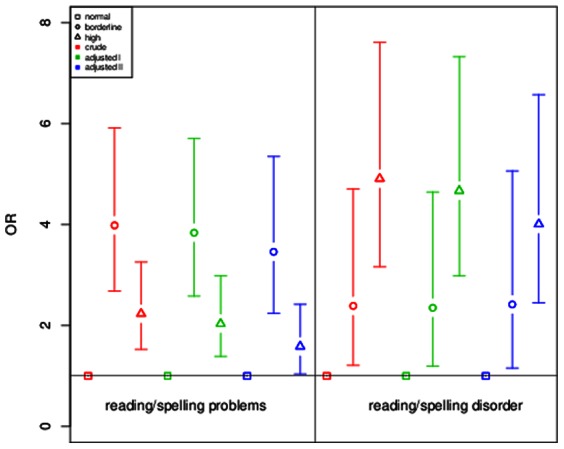
Sensitivity analysis for reading/spelling problems and reading/spelling disorder in GINIplus. ORs and 95% confidence intervals are depicted for reading/spelling problems and reading/spelling disorder in three investigated models (crude, adjusted for gender, age, study and study center and additional adjustment for parental education, age of mother at birth, daily TV/PC use, single parent and ETS exposure). Normal SDQ score was chosen as reference category.

**Figure 2:**
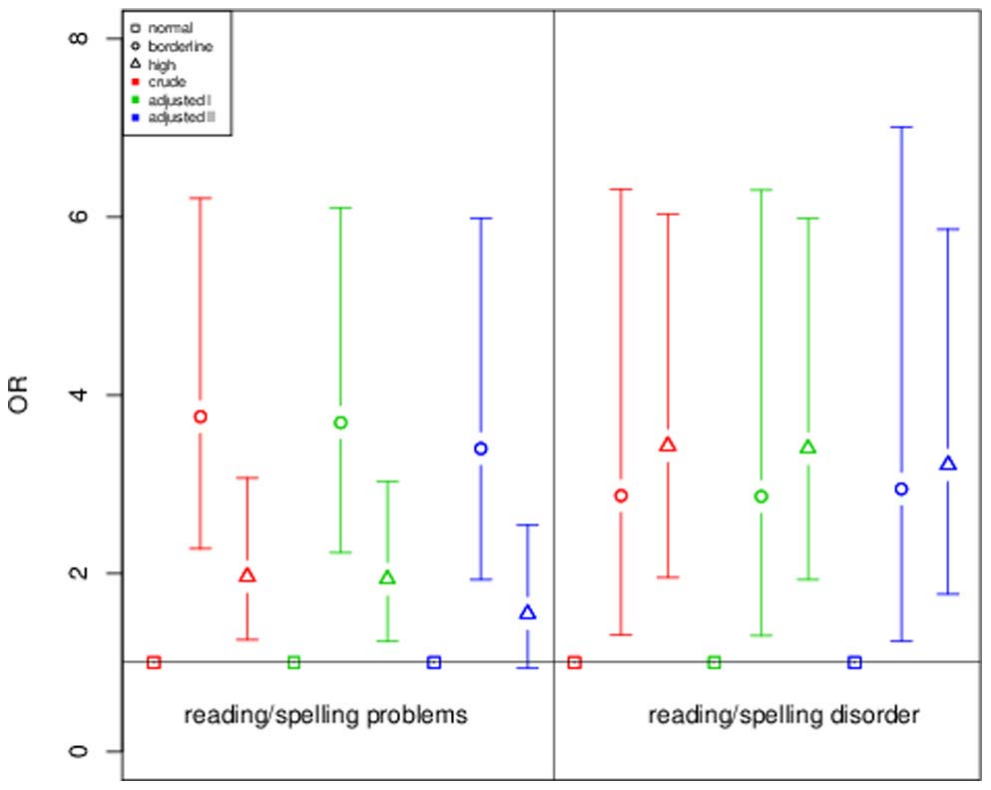
Sensitivity analysis for reading/spelling problems and reading/spelling disorder in males from GINIplus. ORs and 95% confidence intervals are depicted for reading/spelling problems and reading/spelling disorder in three investigated models (crude, adjusted for gender, age, study and study center and additional adjustment for parental education, age of mother at birth, daily TV/PC use, single parent and ETS exposure). Normal SDQ score was chosen as reference category.

**Figure 3:**
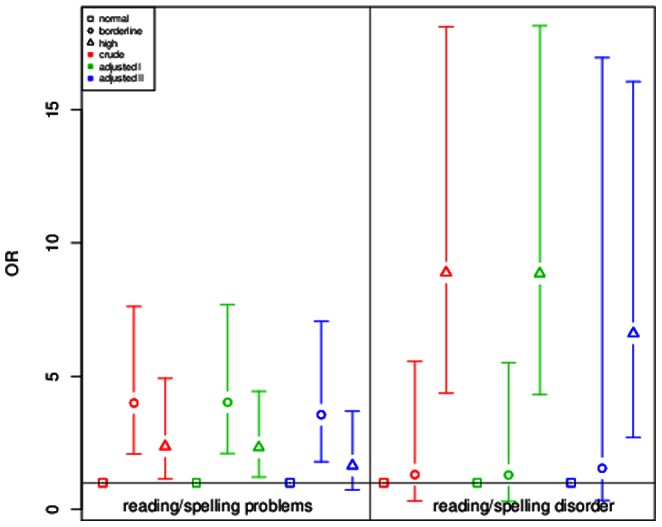
Sensitivity analysis for reading/spelling problems and reading/spelling disorder in females from GINIplus. ORs and 95% confidence intervals are depicted for reading/spelling problems and reading/spelling disorder in three investigated models (crude, adjusted for gender, age, study and study center and additional adjustment for parental education, age of mother at birth, daily TV/PC use, single parent and ETS exposure). Normal SDQ score was chosen as reference category.

In GINIplus, reading/spelling problems and reading/spelling disorder were assessed separately. Therefore a sensitivity analysis on the severity of these symptoms for children included in the GINIplus study was performed (Figure 1). Children with borderline SDQ-score showed a higher OR for having reading/spelling problems (adjusted II model: OR = 3.46, p = 2.32×10^−08^) as compared to children with high SDQ-score (adjusted II model: OR = 1.58, p = 3.26×10^−02^). However, for the risk of showing reading/spelling disorder, results were vice versa: children with high SDQ-score presented with a higher risk for reading/spelling disorder (adjusted II model: OR = 4.00, p = 3.83×10^−08^) as compared to children with borderline SDQ-score (adjusted II model: OR = 2.41, p = 1.94×10^−02^). In the stratified analysis, boys with borderline SDQ-score were at higher risk for presenting with reading/spelling problems (adjusted II model: OR = 3.40, p = 2.25×10^−05^) as compared to boys with high SDQ-score (adjusted II model: OR = 1.55, p = 8.60×10^−02^, Figure 2). For the risk of showing reading/spelling disorder, effects were in the opposite direction: male children with high SDQ-score presented with nearly the same risk (adjusted II model: OR = 3.22, p = 1.32×10^−04^) as compared to males with borderline SDQ-score (adjusted II model: OR = 2.95, p = 1.45×10^−02^). For girls, differences between both groups were more striking (Figure 3): girls with high SDQ-score had a higher risk for showing reading/spelling difficulties (adjusted II model: OR = 6.61, p = 3.10×10^−05^) as compared to girls with borderline SDQ-score (adjusted II model: OR = 1.54, p = 5.72×10^−01^).

In addition to comorbidity between ADHD symptoms and reading/spelling difficulties, we also investigated association between ADHD symptoms and math difficulties. 6.8% of children with ADHD symptoms also presented with math difficulties. Children with ADHD symptoms showed a higher risk of also being affected with math difficulties as compared to children without ADHD symptoms (Table 3). Without adjustment for covariates, the OR was 2.67 (p = 3.95×10^−04^) for children with borderline and 2.87 (p = 3.21×10^−06^) for children presenting with a high SDQ-score. After adjusting for covariates these values were 2.82 (p = 4.90×10^−04^) and 2.55 (p = 3.63×10^−13^) respectively. Stratified analyses for GINIplus and LISAplus showed similar results in both studies (data not shown).

**Table 3:** Association results from logistic regression analysis between math difficulties and ADHD symptoms measured by the SDQ-hyperactivity/inattention score.

SDQ hyperactivity score											
Model	Normal			Borderline				High			
	dyscalculic^1^	non dyscalculic^2^	OR	dyscalculic^1^	non dyscalculic^2^	OR [95% CI]	p-value	dyscalculic^1^	non dyscalculic^2^	OR [95% CI]	p-value
Dyscalculia crude	n = 102	n = 4000	1.00 (reference)	n = 16	n = 235	2.67 [1.55–4.60]	**3.95**×**10** ^−**04**^	n = 26	n = 355	2.87 [1.84–4.48]	**3.21**×**10** ^−**06**^
Dyscalculia adjusted I^3^	n = 102	n = 4000	1.00 (reference)	n = 16	n = 235	3.03 [1.75–5.26]	**7.69**×**10** ^−**05**^	n = 26	n = 355	3.48 [2.20–5.50]	**9.91**×**10** ^−**08**^
Dyscalculia adjusted II^4^	n = 91	n = 3576	1.00 (reference)	n = 15	n = 207	2.82 [1.58–5.07]	**4.90**×**10** ^−**04**^	n = 21	n = 320	2.55 [1.52–4.26]	**3.63**×**10** ^−**04**^

1 refers to the number of individuals with math difficulties for whom full phenotypic information was available and who were included in regression analysis.

2 refers to the number of individuals without math difficulties for whom full phenotypic information was available and who were included in regression analysis.

3 adjusted for gender, age, study and study center.

4 additional adjustment for parental education, age of mother at birth, daily TV/PC use, single parent and ETS exposure.

p-values <0.05 are depicted in bold.

Concerning comorbidity between ADHD symptoms and math difficulties we observed high gender differences. While males with borderline SDQ-score were not significantly at higher risk for math difficulties as compared to males with normal SDQ-score (adjusted II model: OR = 1.82, p = 2.39×10^−01^), males with high SDQ-score had a significantly higher risk (adjusted II model: OR = 2.14, p = 3.70×10^−02^). In the female group, children with borderline SDQ-score as well as with high SDQ-score had a significantly higher risk for math difficulties (borderline SDQ- adjusted II model: OR = 3.75, p = 3.67×10^−04^; high SDQ- adjusted II model: OR = 3.06, p = 3.00×10^−03^).

In studies of comorbidity possible treatment effects have to be considered. Treatment might influence ADHD symptoms as well as reading/spelling and math skills. In the combined GINI/LISA sample, data on therapy with methylphenidate was available for 3,601 children. The substance was administered to 87 (2.4%) out of these individuals. However, including this information as covariate into the analysis did not change the statistical association results for reading/spelling difficulties or for math difficulties (data not shown).

## Discussion

We investigated the association between ADHD symptoms and reading/spelling and math difficulties in a pooled sample consisting of children from the LISAplus and GINIplus studies. Children with ADHD symptoms showed a significantly higher risk for reading/spelling difficulties as well as for math difficulties as compared to children presenting with normal SDQ-scores. Observed effects seem to be triggered by gender: while the prevalence for reading/spelling difficulties was higher in males (14.1%) than in females (8.7%), stratification analysis revealed that females showing ADHD symptoms were at a higher risk for also having reading/spelling difficulties than males. Only males with high SDQ-score were at significantly higher risk for having math difficulties while in females both groups- with borderline and high SDQ- showed significantly higher risk. For interpretation of these results, it has to be noted that we do not have information on which symptoms children presented with first – ADHD or dyslexia/dyscalculia symptoms, i.e. we can neither infer causality nor the chronology of symptoms. To diagnose a child with comorbid ADHD ADHD-symptoms need to be present before reading/spelling or arithmetic problems arised [25]. For this, a thorough medical history is essential.

Up to date, few studies, partly population-based but with smaller sample sizes than our study, reported comorbidity between ADHD and dyslexia [11], [26], [27]. In a longitudinal twin study Greven et al. [28] showed that ADHD and reading significantly predicted each other over time and that this association was mainly due to shared genetic factors. Results on the relationship between ADHD and dyscalculia are less consistent. While Kaufmann et al. [29] report a link between ADHD and numerical processing, Monuteaux et al. [30] suggest that both disorders are distinct. Our results point to a relationship between ADHD and both – dyslexia and dyscalculia. We would assume that for children who suffer from both learning disorders, estimated risk values would be even higher. However, in our sample, only 62 individuals with reading/spelling and math difficulties were available and this number does not allow for valid statistical conclusions.

We observed sex-specific differences in the association between ADHD and reading/spelling difficulties. The scale used for assessment of ADHD symptoms in our study combines hyperactivity and inattention symptoms and is not validated for a separate analysis of these two symptoms. When Willcutt et al. [27] investigated the ADHD core symptoms inattention and hyperactivity/impulsivity stratified by gender, they found that reading disability was significantly associated with inattention in both girls and boys, but with hyperactivity/impulsivity only in boys. Thus, in future studies a more detailed analysis of ADHD subscales might be helpful to enlighten possible gender effects of the comorbidity.

We also found high differences in the effect sizes for associations between ADHD and math difficulties in girls and boys. To our knowledge, in the few studies on ADHD and dyscalculia that have already been published, sex-stratified analysis was not performed.

Our results are based on a large sample size in which prevalences for the different disorders are similar to already reported estimates. Further strengths are related to the population-based characteristic of both studies and to the consistency of results between GINIplus and LISAplus. In addition, we cautiously adjusted for several socio-economic factors such as parental education and TV/PC use to avoid confounding effects. However, there are also some flaws in the study design. First, ADHD symptoms are assessed by the parent-reported SDQ, a screening instrument. No information on physician diagnosed ADHD was available. Furthermore, presence of dyslexia/dyscalculia symptoms is also based on parental questionnaires and validity of diagnosis may therefore exhibit uncertainness. However, self-reported data on spelling and reading have been reported to show high concordance rates with psychometric tests [31].

Only in GINIplus, reading/spelling problems and reading/spelling disorder were assessed separately. The overall results are based on the combined sample exhibiting both – therefore ORs might be overestimated. The same holds true for math difficulties as no distinction between problems in math and dyscalculia was made.

Unfortunately, no data on the psychopathology of the parents is available. We can therefore make no conclusions on a possible aggravation of symptoms in families or on a genetic component of the disorders.

LISAplus and GINIplus are ongoing longitudinal studies and the 15-year-follow up data will again include questions on reading/spelling and math difficulties. This will enable to analyse the long-term development of the disorders and if special training in reading/spelling lead to a decrease in literacy problems.

In our study ADHD symptoms and learning difficulties were comorbid, taking into account possible confounder variables. Several scenarios underlying this comborbidity are thinkable [32]: 1) ADHD implicates the development of learning disorders. 2) Genetic susceptibility factors for ADHD alone are different from risk variants for comorbid ADHD. ADHD alone and comorbid ADHD are thus genetically/environmentally distinct. 3) The comorbidity is due to environmental factors. In future studies it would be very interesting to conduct genome-wide association analyses in children with ADHD only, dyslexia/dyscalculia only as well as in comorbid children. This might enlighten what processes are causing the comorbidity on a genetic level.

From a diagnostic and therapeutic point of view, it is important to not only check for ADHD or learning disorders alone, but rather for ADHD and reading/spelling or math difficulties in parallel and to adapt the training and therapy accordingly. Children with learning disorders who also present with ADHD symptoms should receive different training as compared to children who present only with dyslexia and dyscalculia because ADHD itself can have an impact on the training methods and training success.

### Conclusion

We found that children with ADHD symptoms are at higher risk for reading/spelling and math difficulties compared to children without ADHD symptoms. The exact functional process and causality pattern behind this comorbidity remains unknown.
